# Acute Lateral Ankle Sprain Impairs Function and Strength Without Altering Muscle or Tendon Stiffness: A Controlled Observational Study

**DOI:** 10.1111/os.70082

**Published:** 2025-05-29

**Authors:** Pedro Bainy Franz, José Roberto de Souza Júnior, Hortência Cordeiro de Lima, Estevão de Souza Diniz, Rogério de Brito Aguiar, Jeam Marcel Geremia, Henrique Mansur, Rita de Cássia Marqueti, João Luiz Quagliotti Durigan

**Affiliations:** ^1^ Laboratory of Muscle and Tendon Plasticity Graduate Program in Rehabilitation Science, Faculty of Health Sciences and Technologies, University of Brasília Brasilia Brazil; ^2^ University Center of the Federal District – UDF Brasilia Brazil; ^3^ Exercise Research Laboratory Federal University of Rio Grande do Sul Porto Alegre Brazil; ^4^ Laboratory of Molecular Analysis Graduate Program in Rehabilitation Science, Faculty of Health Sciences and Technologies, University of Brasília Brasilia Brazil; ^5^ Rehabilitation Sciences Program University of Brasilia Brasilia DF Brazil

**Keywords:** Achilles tendon, ankle sprains, elastography, functional performance, muscle strength

## Abstract

**Introduction:**

Acute lateral ankle sprain (LAS) frequently results in persistent functional limitations. Understanding changes in calf muscle and Achilles tendon (AT) stiffness after LAS may shed light on mechanisms underlying impaired function.

**Objective:**

To investigate the effects of acute LAS on the mechanical properties of the calf muscles and the Achilles tendon, ankle function, pain, edema, and strength.

**Methods:**

This controlled observational study was conducted from August 2023 to January 2025. Fourteen participants with acute LAS and 14 healthy controls were evaluated twice, 6 weeks apart. Shear wave elastography (SWE) assessed the stiffness of the triceps surae and AT. Ankle function, pain, and edema were evaluated using the Foot and Ankle Outcome Score, Visual Analog Scale, and figure‐of‐eight method. Plantar flexion strength was measured via isometric dynamometry.

**Results:**

No significant differences in stiffness were found between or within groups (soleus: *p* = 0.932; MG: *p* = 0.760; LG: *p* = 0.800; AT: *p* = 0.070), although a time effect (*p* = 0.005, η^2^ = 0.269) indicated a general increase in AT stiffness over time (MD = −0.72, *p* = 0.05, d = 2.86). At baseline, the LAS group exhibited reduced ankle function (MD = 3.43, *p* < 0.001, d = 2.20), increased pain (MD = 1.88, *p* < 0.001, d = 1.86), and greater edema (MD = −51.27, *p* < 0.001, d = −3.58). Over time, improvements were noted in function (MD = −37.04, *p* < 0.001, d = 2.27), pain (MD = 2.66, *p* < 0.001, d = −1.31), and edema (MD = 1.07, *p* = 0.014, d = −0.95), but ankle function remained lower in the LAS group at follow‐up (MD = −14.17, *p* < 0.001, d = −1.79). For plantar flexion strength, no group × time interaction was found (*p* = 0.745), but a group effect indicated lower peak torque in the LAS group (MD = −32.05, *p* = 0.012, d = −3.82). A time effect (*p* < 0.001, η^2^ = 0.622) showed increased torque across both groups (MD = −18.74, *p* < 0.001, d = 3.07).

**Conclusion:**

LAS reduces ankle function and leads to pain and edema but does not induce notable changes in calf muscle or AT stiffness within 6 weeks.

## Graphical abstract

This study used shear‐wave elastography to assess Achilles tendon and calf muscle stiffness following acute lateral ankle sprain (LAS). Despite the expected alterations due to injury and unloading, no significant stiffness changes were observed over 6 weeks. Findings suggest potential compensatory mechanisms preserving tissue mechanical properties post‐LAS.

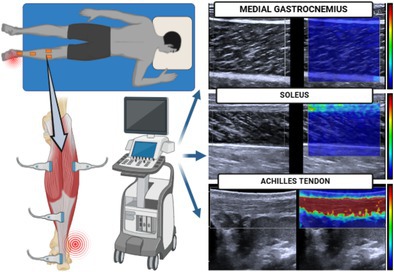

## 1 Introduction

Lateral ankle sprain (LAS) is one of the most prevalent acute musculoskeletal injuries worldwide [1, 2] and ranks among the leading orthopedic injuries of the ankle‐foot complex requiring medical attention [3, 4]. An immediate consequence of LAS is disuse due to pain, joint edema, and reduced weight‐bearing, leading to muscle atrophy, weakness, and impaired function [5, 6] Although conservative treatment protocols for LAS typically last 4 to 6 weeks [7] (Mansur et al. 2022), significant reductions in the cross‐sectional volumes of ankle muscles and tendons have been reported at this time point [7]. Persistent joint laxity [8], low function, and reduced muscle and tendon thickness can endure for years following LAS [7, 9, 10, 11, 12]. While ligaments may heal within 6 weeks after mild or moderate LAS [13], most patients require longer periods to regain pre‐injury function [7, 14, 15, 16].

Muscle disuse, such as that following a lateral LAS, leads to significant neuromuscular consequences. Notably, muscle strength declines more rapidly than muscle thickness during disuse, with strength losses occurring disproportionately early after the onset of disuse [16, 17]. Disuse affects not only the contractile components of musculotendinous structures but also their mechanical and material properties [18, 19]. Previous studies have demonstrated reductions of up to 30% in tendon stiffness and increased hysteresis in knee extensor tendons after 20 days of disuse [18, 20]. Stiffness reflects muscle and tendon mechanics [21] and aids in assessments of tissue health and injury recovery [22, 23]. Importantly, decreased tendon stiffness due to disuse occurs independently of morphological alterations, such as changes in tendon cross‐sectional area [24] and may emerge within as little as 14 days of limb suspension [25].

Additionally, impaired postural control is a well‐documented consequence of both acute and chronic ankle sprains [5, 26]. Understanding whether acute LAS leads to measurable changes in the mechanical properties of calf muscles and the Achilles tendon is essential since these tissues may play a relevant role in joint stability and postural control [27, 28, 29, 30]. The passive stiffness of the calf muscle–tendon complex contributes significantly to ankle joint impedance, especially in conditions requiring postural control and rapid torque production [27, 28, 29, 31]. Loram and colleagues [27, 32] highlighted the importance of Achilles tendon stiffness for ankle joint stiffness and stability but noted that its series elastic contribution is limited, with passive calf muscle stiffness playing a relevant role as well. Other studies support the link between tendon and muscle mechanical properties and joint behavior, showing positive associations between medial gastrocnemius and Achilles tendon stiffness and passive ankle joint stiffness [31]. Similarly, changes in the mechanical stiffness of soft tissues around the ankle can alter joint kinematics and contribute to symptoms following lateral ankle sprain, potentially leading to chronic ankle instability [33, 34]. These findings suggest that alterations in tendon properties may significantly influence joint behavior and stability following injury. However, research on the immediate effects of LAS on tendon and muscle stiffness, as well as their recovery over time, remains scarce.

Changes in muscle and tendon stiffness can be reliably analyzed using ultrasound shear wave elastography (SWE), which offers a non‐invasive method for assessing tissue stiffness without requiring muscle contractions [21, 35, 36, 37, 38, 39]. This technique offers the advantage of assessing stiffness in various tendon [38, 40] and muscle positions, making it particularly useful for individuals with impairments or those unable to contract their muscle [41, 42]. In contrast, methods like Young's modulus rely on physical effort [43], limiting their feasibility for individuals with impairments or those unable to contract their muscles [44]. Given the lack of studies on calf muscles and Achilles tendon (AT) stiffness after LAS, investigating these potential changes over time may help advance our understanding of the impact of LAS on tendon and muscle function and provide indirect but valuable insights into disuse‐related changes in the assessed muscles and tendons. Thus, the current study aimed to investigate the effects of acute LAS on the mechanical properties of the calf muscles and AT in conjunction with ankle function, pain, edema, and strength compared to a paired control group over a six‐week period. We hypothesized that the time since LAS would affect calf muscles and AT mechanical properties, with the six‐week period leading to increased calf muscle stiffness [45, 46, 47, 48] and decreased AT stiffness [19, 22].

## 2 Methods

### 2.1 Study Design

This is a Level 3 longitudinal and observational study with a six‐week follow‐up. The study was approved by the Institutional Review Board at the University of Brasília (number 68997923.2.0000.8093). Informed consent was obtained in accordance with the Helsinki Declaration and local resolution.

### 2.2 Participants

**FIGURE 1:**
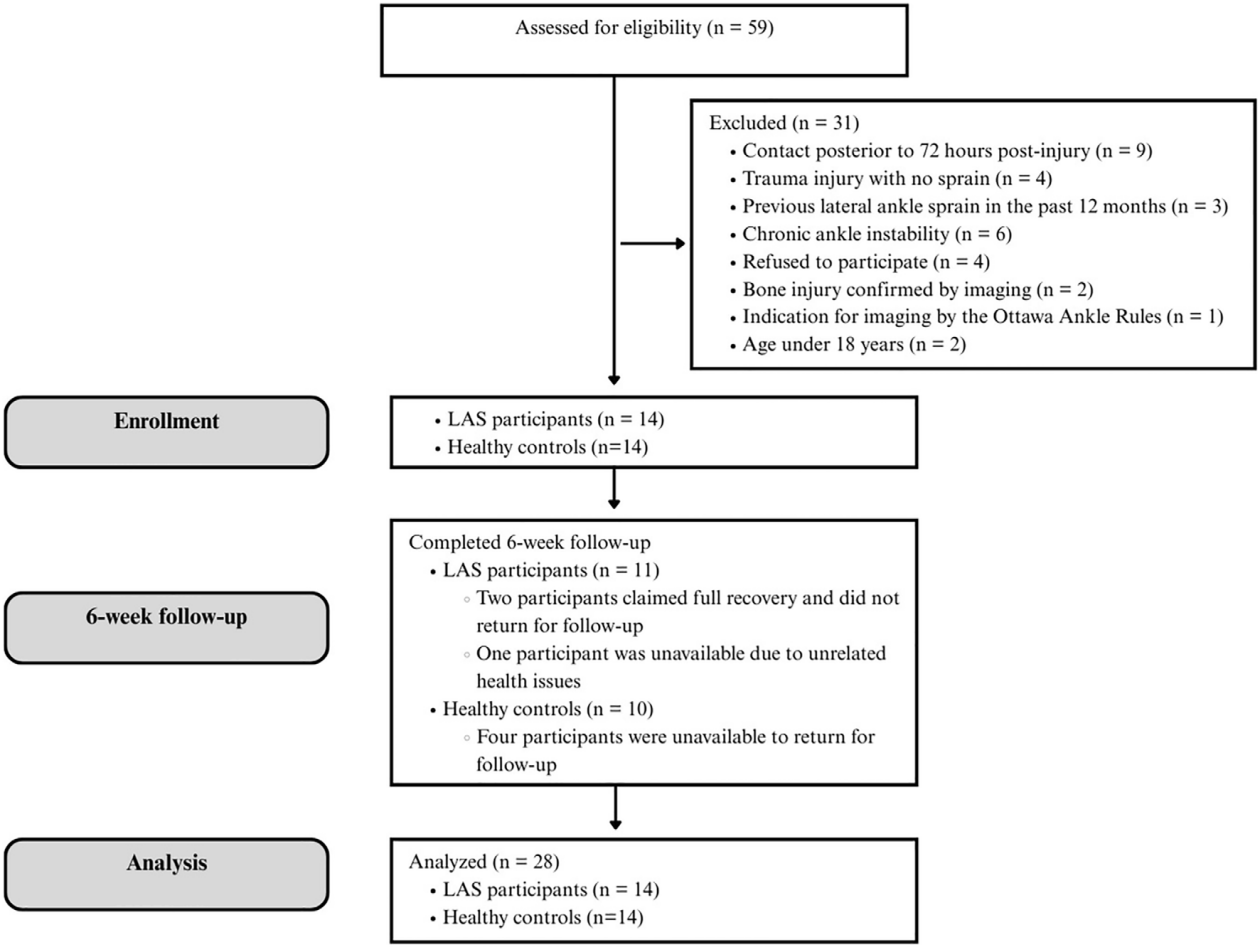
STROBE flowchart. STROBE, Strengthening the Reporting of Observational Studies in Epidemiology.

**FIGURE 2:**
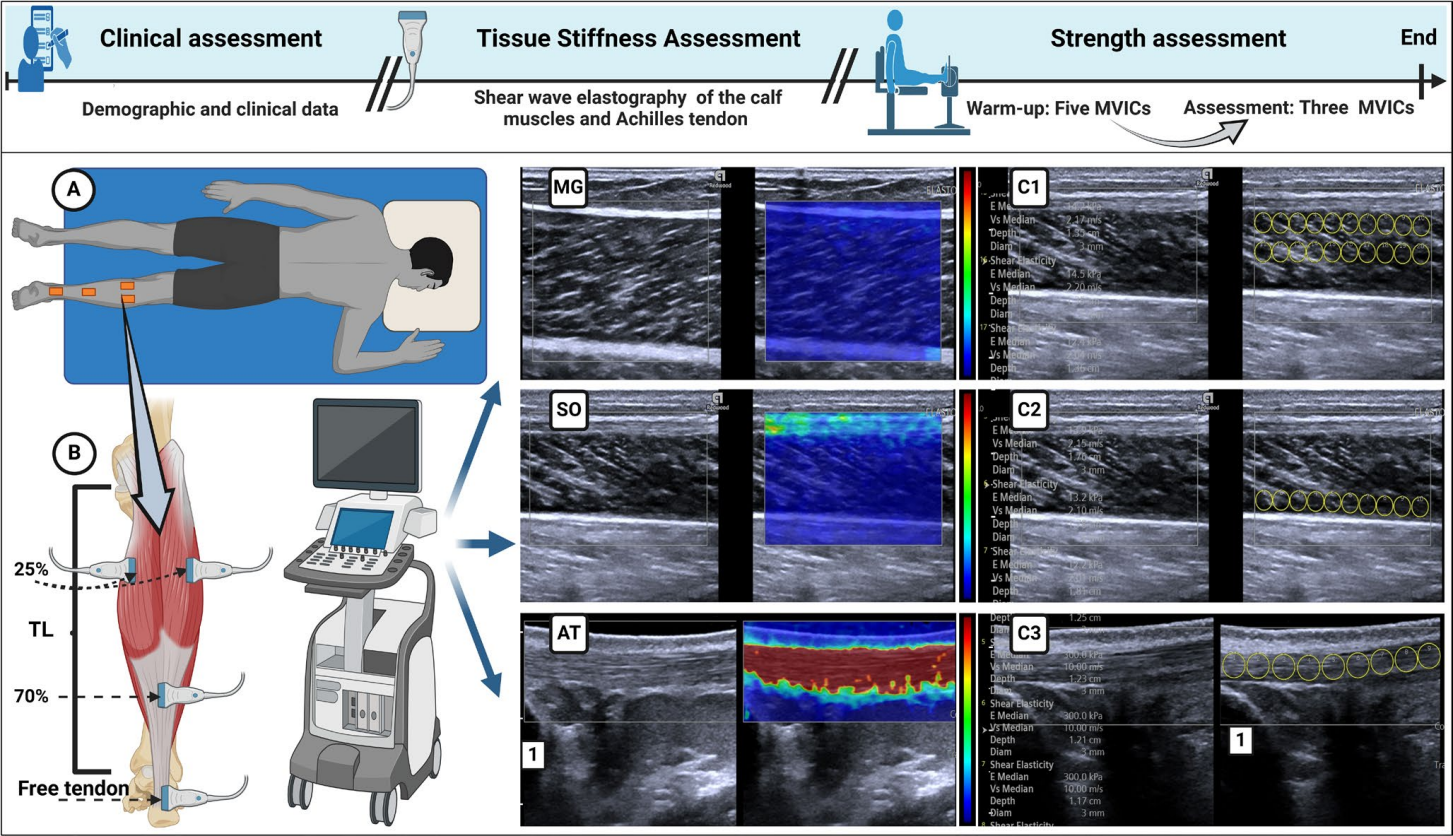
Data collection protocol. Participants underwent three assessments: (1) Clinical Assessment, including demographics, injury history, functional outcomes, edema, and pain; (2) Tissue Stiffness Assessment, using shear wave elastography (SWE) on the soleus (SO), medial gastrocnemius (MG), lateral gastrocnemius (LG), and Achilles tendon (AT), where red indicates more stiffness and blue less stiffness; and (3) Strength Assessment, measuring maximal voluntary isometric contraction (MVIC) of the plantar flexors with an isometric dynamometer. Assessments were conducted at baseline and after 6 weeks. (A) Patient positioning for SW Participants lay prone with the foot relaxed and hanging off the table. (B) Anatomical landmarks: MG and LG at 25% of tibial length (TL), SO at 70% TL, and AT in the free tendon region. TL was measured from the medial tibial plateau to the distal medial malleolus. Muscle stiffness was calculated from 30 regions of interest (ROIs) per scan, averaged across three scans. Due to equipment limits, scans used 20 ROIs (C1) and 10 additional ROIs (C2). Achilles tendon stiffness was measured using 9 ROIs per scan (C3), with three scans averaged. To ensure unbiased analysis, color elastograms were removed before ROI selection, preventing qualitative bias in stiffness interpretation.

Patients with LAS and healthy controls were selected (Figure 1). The inclusion criteria for the LAS patients were: (I) Age between 18 and 60 years and (II) history of grade I or II lateral ankle sprain (LAS) within 72 h prior to evaluation. Grade I represents mild stretching without instability; Grade II (moderate) involves a partial rupture with mild instability; Grade III (severe) is characterized by a complete rupture with significant instability [49, 50]. Participants were excluded if they presented with at least one of the following: chronic ankle instability, another ankle sprain in the past year, a grade III injury (diagnosed via clinical tests or imaging), bone injury confirmed by imaging, or, if no imaging had been taken, an indication for imaging based on the Ottawa Ankle Rules [51], lower limb injuries, or prior ankle surgeries. Healthy controls with no history of ankle sprains, severe lower limb injuries, or ankle, foot, or AT pain in the previous 6 months were included. Control group participants were matched according to sex, age, height, body mass, and body mass index. Both groups underwent identical evaluations twice, 6 weeks apart (Figure 2).

### 2.3 Outcomes

The primary outcome was the mechanical properties of the calf muscles and AT. Secondary outcomes included ankle function (measured by the Foot and Ankle Outcome Score—FAOS), pain, edema, and muscle strength of the plantar flexors, providing clinical context to clarify the extent of disuse in participants with acute LAS. These measures complemented the mechanical assessments, offering a broader understanding of post‐injury impairments.

### 2.4 Experiment Outline

The study was conducted at the Laboratory of Muscle and Tendon Plasticity (LaPlasT) at the University of Brasilia. Participant recruitment and data collection occurred between August 2023 and January 2025. At the beginning of the experimental session, the body mass and height of the participants were recorded. Next, injury history, ankle function, pain, and edema were assessed. The translated and validated FAOS questionnaire was used to assess ankle function [52] across five subscales: pain, symptoms, sports/recreation, daily activities, and quality of life. Participants rated their symptoms on a 0–4 scale, with scores normalized from 0 (extreme) to 100 (none). Pain intensity was measured using the Visual Analog Scale (VAS) as used by Terrier et al. [53], on a 10 cm horizontal line ranging from 0 (“no pain”) to 10 (“severe pain”). Participants were instructed to mark their perceived pain level at the time of assessment. Edema was quantified using the figure‐of‐eight method, adapted from Devoogdt et al. [54], with the zero point at the distal edge of the medial malleolus. Seated participants kept their foot relaxed. The tape followed a path from the medial malleolus, crossing laterally over the ankle to the fifth metatarsal, looping under the arch to the first metatarsal, passing over the lateral malleolus, wrapping around the Achilles tendon, and returning to the start.

Following these clinical assessments, anatomical landmarks to measure tibia length were identified and marked on the participant's skin using a dermographic pencil to ensure consistency in the SWE measurements. The variables measured were the mechanical properties of the soleus (SO), medial gastrocnemius (MG), lateral gastrocnemius (LG), and Achilles tendon (AT).

Lastly, plantar flexion strength was measured using an isometric dynamometer (IsoSystem 2.0, Cefise, SP, Brazil). Participants were seated with their assessed foot secured to the footplate. The hip was positioned at approximately 120°, the knee was fully extended, and the ankle was positioned at approximately 90°. Testing began with a familiarization consisting of three maximal voluntary isometric contractions (5 s each), with a 2‐min rest between contractions. Following familiarization, three maximal voluntary isometric contractions (MVICs) were recorded using the same protocol. The average peak torque from these trials was used for data analysis. The details of the mechanical property assessments are provided below. The procedures followed during the experimental session are presented in Figure 2.

### 2.5 Testing Protocol for Muscle and Tendon Mechanical Properties

The mechanical properties of the calf muscles AT were evaluated through stiffness measurements using SWE. Muscle stiffness in the SO, MG, LG, and AT was measured with the ACUSON Redwood Ultrasound System (Siemens Healthineers, Erlangen, Germany) and a linear probe (10–L4 MHz). Musculoskeletal and tendon presets were applied with a 0–10 m/s (0–300 kPa) scale. For muscle assessments, settings included a smoothing level of 3, a gain of 9 dB, and persistence of 3. Tendon measurements used the same settings, except for a gain of −3 dB. SWE measurements were obtained with participants lying prone, knee fully extended, and ankle relaxed and hanging off the table in a neutral position [55]. The ultrasound transducer was covered with water‐soluble gel. A large rectangular field of view (FOV) captured the entire target tissue in the B‐mode image [56], with 3 mm circular regions of interest (ROIs) used for shear wave speed measurements. To account for tissue anisotropy, the probe was positioned perpendicular to the target [56]. Assessments were conducted in a climate‐controlled room (23°C–25°C) to prevent temperature‐related stiffness changes [57]. For muscle assessment, the probe was placed longitudinally along the muscle fibers and perpendicular to the skin at 25% (LG and MG) and 70% (SO) of the tibial length, measured proximally to distally [55]. The tibial length was defined as the distance from the medial tibial plateau to the distal medial malleolus [55].

SWE scans were performed at the thickest muscle region, identified using a transverse B‐mode image. The probe was then aligned parallel to the orientation of the muscle fascicles, ensuring that multiple fascicles were continuously visible [58]. Thirty ROIs were manually distributed among the superficial, intermediate, and deep layers of the evaluated muscle, ensuring that no ROI contacted the superficial or deep aponeuroses to minimize their influence as artifacts on the measurements. For tendon assessment, the AT was evaluated using a probe positioned on the tendon, aligned parallel to its orientation in the free tendon area [22]. To reduce the influence of bone tissue on SWE data [59], measurements were taken where the AT overlies Kager's fat pad. Nine regions of interest (ROIs) were selected along the tendon length, avoiding the area overlapping with the calcaneus. Probe orientation was verified by ensuring homogeneous visibility of the tendon and peritendon structures in the B‐mode ultrasound image. A gel pad (Hill Laboratories) was used to ensure acoustic coupling without applying additional pressure. To blind the assessor from qualitative information about tissue stiffness before distributing the ROIs, the SWE color elastogram was removed from the image before execution and reapplied after data acquisition for both muscle and tendon SWE (Figure 2). SWE velocity was used instead of shear wave modulus to minimize inaccuracies in Young's modulus estimation, as the latter can be influenced by assumptions about tissue density and equipment limitations [60]. Each tissue was evaluated three times. For each analysis, the shear wave speed for a participant was calculated as the mean of 30 regions of interest (ROIs) for muscles and 9 ROIs for the AT. The overall tissue stiffness was determined as the average of the three analyses.

### 2.6 Reliability Assessment

SWE was conducted by two physical therapist researchers, both trained in musculoskeletal ultrasound imaging and standardized SWE assessment protocols. To assess the reliability of the SWE measurements, intra‐ and inter‐rater reliability were evaluated in the same session in a selected subset of healthy participants (*n* = 12). For intra‐rater reliability, the same rater performed three SWE measurements on each participant. For inter‐rater reliability, a second rater, blinded to the first rater's results, performed three further independent SWE assessments 10 min after the first rater had completed their evaluation. To eliminate bias, all skin markings were erased between measurements, and the rater remeasured tibial length (TL) and reidentified anatomical landmarks to ensure an independent reassessment.

### 2.7 Sample Size

A post hoc sample size calculation was conducted using the partial eta‐square value (η^2^) of the group‐by‐time interaction for shear‐wave velocity of the Achilles tendon (η^2^ = 0.121) obtained after data collection from 28 subjects. In G*Power software, we performed repeated‐measures ANOVA (within‐between interaction) with the following parameters: effect size f = 0.37 (obtained using η^2^ = 0.121); level of significance α = 0.05; power = 95%; number of groups = 2 (LAS vs. control); number of measurements = 2 (baseline vs. six‐weeks). The G*Power software used the effect size index (f) for this analysis. The effect size f was directly calculated from the η^2^ through the following formula: *f = p η* ^2^/*(1* − *η* ^2^ *)*. The analysis indicated that a sample size of 26 participants would be sufficient for the study.

### 2.8 Data Analysis

Data were analyzed using SPSS (Statistical Package for the Social Sciences) version 26.0. Descriptive statistics consisted of means and standard deviations for continuous variables and frequencies and percentages for categorical variables. Data normality was tested using the Shapiro–Wilk test, while homogeneity of variances was tested using Levene's test. Missing data were imputed for both groups using the maximization method. Independent t tests were used to compare the general participants characteristics (age, body mass, height and body mass index) across study groups. A chi‐square test was used to compare the proportion of male and female participants across the study groups. Group × time interactions for the primary and secondary outcomes were calculated using Two‐way ANOVAs (group × time). Group (acute lateral ankle sprain × control) was used as the independent factor, time (baseline × six‐weeks) as the repeated factor, and the primary (clinical aspects) and secondary outcomes (shear‐way velocity and peak torque) as dependent variables. The Bonferroni's post hoc test was applied for pairwise comparisons. In the absence of a group × time interaction, the group and time effects were presented. A significance level of *p* < 0.05 was used for all analyses. Effect sizes were determined using partial eta‐squared (η^2^), where values of η^2^ > 0.01 were defined as small, η^2^ > 0.06 as medium, and η^2^ > 0.14 as large [60] (Cohen 1988). Cohen's d (d) was used for the pairwise comparisons. Values of d = 0.2 were defined as small, d = 0.5 as medium, and d = 0.8 as large [61] (Cohen 1988). The reliability of shear‐wave velocity measurements for the calf muscles and Achilles tendon was assessed using the Intraclass Correlation Coefficient (ICC). A mixed two‐way model with absolute agreement was applied. Intra‐rater reliability was determined based on three repeated measurements (k = 3), while inter‐rater reliability was calculated by comparing the average of three measurements from each of the two raters (k = 2). ICC values were interpreted as follows: < 0.50, poor reliability; 0.51–0.75, moderate reliability; 0.76–0.90, good reliability; and > 0.91, excellent reliability [62].

## 3 Results

### 3.1 General Characteristics

The study included 28 participants (18 females, 10 males), divided into two groups: (I) Acute lateral ankle sprain (LAS) (*n* = 14) and (II) healthy controls (*n* = 14) (Figure 1). Participants had a mean age of 29.03 ± 8.07 years, body mass of 73.25 ± 19.30 kg, height of 1.69 ± 0.09 m, and BMI of 25.26 ± 5.07 kg/m^2^. No significant between‐group differences were found (all *p* > 0.05) (Table 1).

**TABLE 1:** General characteristics of the participants (*n* = 28).

	LAS (*n* = 14)	Healthy (*n* = 14)	MD (IC 95%)	*p*
Sex				
Female	10 (71.4%)	8 (57.1%)	—	0.430
Male	4 (28.6%)	6 (42.9%)		
Age, years	29.57 ± 10.16	28.50 ± 5.61	−5.30 to 7.44	0.366
Body mass, kg	71.71 ± 20.11	74.78 ± 19.07	−18.30 to 12.16	0.267
Height, m	1.68 ± 0.09	1.70 ± 0.10	−0.09 to 0.05	0.341
BMI, kg/m^2^	25.09 ± 5.52	25.42 ± 4.77	−4.34 to 4.68	0.434

*Note*: Data presented as count and percentage or mean ± standard deviation.

Abbreviations: 95% CI, confidence intervals of 95%; BMI, body mass index; MD, mean difference.

### 3.2 Muscle and Tendon Mechanical Properties

Intra‐rater reliability of shear‐wave velocity measurements demonstrated results varying from good (0.76–9.90) to excellent (> 0.91) for both examiner 1 (E1) and examiner 2 (E2) for the SO (E1 ICC = 0.827; E2 ICC = 0.954), MG (E1 ICC = 0.945; E2 ICC = 0.929), LG (E1 ICC = 0.888; E2 ICC = 0.947), and AT (E1 ICC = 0.846; E2 ICC = 0.926). Inter‐rater reliability was good for the soleus (ICC = 0.807), medial gastrocnemius (ICC = 0.905), lateral gastrocnemius (ICC = 0.867), and Achilles tendon (ICC = 0.880). No significant group × time interactions were found for shear‐wave velocity of the calf muscles and Achilles tendon (all *p* > 0.05) (Table 2). However, a time effect for the shear‐wave velocity of the Achilles tendon was found (*p* = 0.005, η^2^ = 0.269). Higher stiffness was found at the six‐week assessment (MD = −0.72, −1.21 to −0.24, *p* = 0.05, d = 2.86).

**TABLE 2:** Differences in muscle and tendon stiffness, pain, edema, ankle function, and peak torque (*n* = 28).

Muscle/Tendon	Groups	Baseline	6‐weeks	*p*	η^2^
Soleus (m/s)	LAS	2.14 ± 0.37	2.05 ± 0.25	0.932	< 0.001
	Healthy	2.35 ± 0.48	2.27 ± 0.40		
Medial Gastrocnemius (m/s)	LAS	2.10 ± 0.19	2.06 ± 0.23	0.760	0.004
	Healthy	2.21 ± 0.31	2.21 ± 0.36		
Lateral Gastrocnemius (m/s)	LAS	1.92 ± 0.22	1.94 ± 0.21	0.800	0.003
	Healthy	2.06 ± 0.20	2.06 ± 0.29		
Achilles Tendon (m/s)	LAS	8.71 ± 1.01	9.00 ± 0.63	0.070	0.121
	Healthy	8.26 ± 1.94	9.43 ± 1.43		
Pain, 0–10 (cm)	LAS	3.42 ± 2.21^a^, ^b^	0.76 ± 1.84^a^	0.003*	0.298
	Healthy	0.00 ± 0.00^b^	0.15 ± 0.06		
Edema (cm)	LAS	1.87 ± 1.5^a^	0.79 ± 0.55^a^	0.017*	0.199
	Healthy	−0.01 ± 0.28^b^	0.38 ± 1.55		
FAOS (0–100)	LAS	47.49 ± 20.22^a^, ^b^	84.53 ± 11.06^a^, ^b^	< 0.001*	0.625
	Healthy	98.76 ± 1.43^b^	98.71 ± 1.82^b^		
Peak Torque (N m)	LAS	58.91 ± 30.98	78.60 ± 39.06	0.745	0.004
	Healthy	91.91 ± 26.93	109.71 ± 31.10		

*Note*: Data presented as mean ± standard deviation. LAS (*n*‐ = 14) and Healthy (Control) (*n* = 14) groups; *Group × time interaction—*p* < 0.05.

Abbreviation: FAOS, Foot and Ankle Outcome Score.

^a^ Within‐group differences.

^b^ Between‐group differences.

### 3.3 Clinical Outcomes

Significant group × time interactions were found for pain (*p* = 0.003, η^2^ = 0.298), edema (*p* = 0.017, η^2^ = 0.199), and ankle function (*p* < 0.001, η^2^ = 0.625) (Table 2). At baseline, the LAS group presented significantly greater levels of pain (MD = 3.43, 2.21 to 4.64, *p* < 0.001, d = 2.20) and edema (MD = 1.88, 1.03 to 2.72, *p* < 0.001, d = 1.86), and worse ankle function (MD = −51.27, −62.41 to −40.13, *p* < 0.001, d = −3.58) compared to the control group. At the six‐week assessment, only ankle function remained significantly different between groups, with lower levels persisting in the LAS group (MD = −14.17, −20.33 to −8.02, *p* < 0.001, d = −1.79).

Also, the LAS group experienced significant improvements in pain (MD = 2.66, 1.49 to 3.84, *p* < 0.001, d = −1.31) edema (MD = 1.07, 0.23 to 1.91, *p* = 0.014, d = −0.95), and ankle function (MD = −37.04, −45.24 to −28.84, *p* < 0.001, d = 2.27) at the 6‐week assessment compared to baseline.

### 3.4 Peak Torque

No significant group × time interaction was found for the peak torque of the calf muscles (*p* > 0.05) (Table 2). However, time (*p* < 0.001, η^2^ = 0.622) and group effects (*p* = 0.012, η^2^ = 0.219) were found for this outcome. Higher peak torque was found at the six‐week assessment (MD = −18.74, −24.63 to −12.856, *p* < 0.001, d = 3.07). In addition, higher peak torque was found in the control group (MD = −32.05, −56.47 to −7.64, *p* = 0.012, d = −3.82).

## 4 Discussion

To our knowledge, this is the first study to comprehensively investigate the impact of acute LAS on the mechanical properties of calf muscles and the AT, along with evaluations of ankle function, strength, and pain. Contrary to our hypothesis, we found no significant differences in calf muscle or AT stiffness between individuals with acute LAS and controls. This unexpected finding suggests that the functional deficits observed in the LAS group may not be primarily attributable to alterations in tissue stiffness. Our results suggest that muscle and tendon tissues may adapt to injury by maintaining mechanical properties that can support function despite damage due to LAS. Clinicians should consider injury grade, time since sprain, and loading conditions during rehabilitation, as these factors affect the calf muscles and AT. Rehabilitation strategies emphasizing balanced ankle loading and even strain distribution may optimize recovery, even in the absence of immediate tissue stiffness changes detected by SWE.

### 4.1 Ankle Function, Pain, Edema and Strength

Tendons and muscles typically exhibit distinct responses to disuse and injury [18, 19]. Muscle stiffness can increase due to a relative rise in extracellular matrix content following muscle atrophy [45, 46, 47, 48], while tendons stiffness may decrease due to reduced loading [19, 22]. However, despite the presence of functional deficits following acute LAS, our findings did not reveal notable changes in calf muscle or AT stiffness within the study period. Several factors could explain this dissociation. First, neuromuscular and sensorimotor impairments, such as altered muscle activation patterns and decreased proprioception, are known consequences of LAS [11, 14, 63]. These impairments can significantly contribute to functional limitations, even in the absence of detectable changes in tissue stiffness. Second, pain itself can inhibit muscle function and alter movement patterns [64]. The observed pain in the LAS group, even at the six‐week follow‐up, could contribute to functional deficits, independent of tissue stiffness. Finally, psychological factors, such as fear‐avoidance beliefs, can play a role in recovery after LAS [16]. These factors can influence an individual's willingness to engage in activities and, consequently, their functional outcomes. Therefore, rehabilitation programs for lateral ankle sprains should address these multifaceted influences, including neuromuscular retraining, pain management, and psychological support, in addition to addressing any potential structural adaptations.

### 4.2 Muscle and Tendon Mechanical Properties

One possible explanation for our findings is that the severity of the injury plays a critical role in the extent of tissue adaptations. Participants with grade I and II injuries typically maintain some degree of functional activity and loading [5, 13] while grade III injuries often demand prolonged immobilization and more restrictive rehabilitation protocols, conditions that are more likely to induce measurable changes in muscle and tendon stiffness due to extended periods of disuse and altered loading [5]. Accordingly, Mansur et al. [7] found significant reductions in the cross‐sectional area and volumes of ankle muscles and tendons after 6 weeks of grade II and III acute LAS. This suggests that substantial structural adaptations may require more severe injury conditions than those in our study population. Consequently, the exclusion of participants with grade III LAS may have limited our ability to detect stiffness changes using SWE.

Since changes in muscle stiffness due to disuse are closely linked to changes in muscle mass [48], our baseline assessment, conducted within 72 h post‐injury, aimed to measure stiffness when small to no morphological alterations could have occurred [17, 65]. This early evaluation established a baseline level for tracking changes over time. Short‐term studies suggest muscle mass loss may occur within 2 to 4 days of limb disuse [17, 65], but measures of human muscle have described increased muscle stiffness following 60 days of bed rest [48]. In the same direction, tendons exhibit a high degree of plasticity in response to mechanical loading, unloading, and injury [19]. Prolonged unloading, such as complete limb suspension or bed rest from 14 to 23 days, has been shown to reduce tendon stiffness, likely due to decreased collagen synthesis and altered matrix organization [18, 25]. However, partial loading may mitigate these effects, as even minimal mechanical stimuli seem sufficient to maintain tendon homeostasis [66, 67]. Christensen et al. [66] examined how calf muscle, AT cross‐sectional area, and tendon collagen synthesis responded to 2 weeks of leg suspension followed by a two‐week rehabilitation period. The authors reported no changes in tendon cross‐sectional area at any time point, a reduction in muscle cross‐sectional area after suspension, and full muscle cross‐sectional area recovery after rehabilitation. In the current study, while 72 h may not have been enough for the onset of stiffness changes in our studied population, the following 6 weeks of disuse or partial loading experienced by our participants may have allowed for compensatory mechanisms that helped maintain tissue properties within normal ranges.

Despite the growing research on SWE for assessing muscle and tendon conditions, no previous studies were found that examined the effects of LAS. Previous studies on muscle and tendon stiffness have shown varying results depending on the disuse condition. Zhang et al. [68] and Yoshida et al. [69] observed increased stiffness in some muscles in individuals with medial tibial stress syndrome and MG injuries, respectively. In contrast, Wang et al. [70] found reduced stiffness in sarcopenia. For AT injuries, studies by Chen et al. [71] and Frankewycz et al. [72] reported lower stiffness in ruptured ATs and post‐repair tendons. Additionally, Zhang et al. [73] and Busilacchi et al. [74] found a positive correlation between (SWE) and functional outcomes, while lower AT stiffness has been consistently noted in tendinopathy studies [75, 76, 77, 78, 79].

Given the lack of SWE studies on calf muscles and the Achilles tendon following LAS, our results offer indirect yet valuable insights into disuse‐related changes in these muscles and tendons. This is particularly relevant when comparing our findings to those of Kawai et al. [80] and McPherson et al. [81], both of whom found no differences in passive muscle stiffness at rest between injured and control groups post‐surgery. On the other hand, McPherson et al. [81] observed higher vastus lateralis stiffness at 12 months post‐ACL surgery, suggesting that time and injury type may influence muscle stiffness recovery. Our results are consistent with the findings of Kawai et al. [80] and McPherson et al. [81], who reported no differences in passive stiffness between groups, despite some methodological differences in muscle and skeletal muscle lesion assessments. This comparison underscores the significance of considering both passive and active tissue properties when interpreting SWE data in musculoskeletal injuries. Factors like ankle positioning and muscle contraction significantly influence AT and calf muscle stiffness measurements [82]. This limitation is particularly relevant in acute injuries, such as LAS, where assessments were conducted within 72 h post‐injury. Pain, swelling, and functional limitations during this phase restrict the feasibility of performing measurements under varying joint positions or loading conditions. Taken together, our data suggest the need to establish normative SWE values following LAS, enabling future comparisons with healthy individuals, elite athletes, and different muscle injuries.

### 4.3 Limitations and Future Prospects

Our study has limitations, including the inability to perform SWE measurements during active muscle contractions and the exclusion of participants with grade III LAS. Another factor influencing our results is the heterogeneity in managing participants with LAS, particularly regarding injury severity (Grades I and II), professional care, early rehabilitation, and immobilization strategies. Individuals with grade II LAS are more likely to experience prolonged immobilization and greater functional limitations compared to those with grade I injuries [5], potentially leading to more pronounced tissue alterations. However, variability in rehabilitation protocols, ranging from early mobilization and functional exercises to rigid immobilization, may have introduced inconsistent mechanical stimuli, affecting tissue adaptation [19]. Additionally, our SWE equipment has a maximum measurement limit of 300 kPa (10 m/s), and multiple tendon measurements reached this upper threshold. Although injured AT typically exhibits SWE values below this limit [72, 76], it could be suggested that the actual tendon stiffness values may have exceeded 10 m/s but were not able to be distinguished within the device's range. This limitation has been previously described in the literature [60, 83, 84], and potential confounding factors that may influence tendon strain should be considered in future studies.

From a rehabilitation perspective, our findings add to the growing evidence that clinical discharge after a standard 4–6‐week program may not indicate full structural or functional recovery. Because changes in stiffness were not the main limiting factor, rehabilitation should focus on neuromuscular retraining, strength restoration, and progressive loading strategies to optimize functional outcomes. While shear wave elastography (SWE) is not yet widely adopted in clinical settings, it shows promise as a tool for monitoring rehabilitation progress. When early active assessments are limited by pain or impairment, SWE offers objective markers of tissue recovery. Integrating it into rehabilitation can complement traditional measures and improve clinical decision‐making.

## 5 Conclusion

LAS significantly reduces ankle function and leads to pain and edema but does not appear to induce notable changes in calf muscle or Achilles tendon stiffness within 6 weeks. Future research should investigate the dynamic mechanical properties of these tissues during activity, explore the influence of standardized rehabilitation protocols, and examine long‐term adaptations following LAS.

## Author Contributions

**Pedro Bainy Franz:** investigation, conceptualization, methodology, data curation, project administration, writing – original draft, writing – review and editing, visualization, validation, formal analysis. **José Roberto de Souza Júnior:** methodology, investigation, data curation, writing – original draft, writing – review and editing, conceptualization, formal analysis, validation. **Hortência Cordeiro de Lima:** conceptualization, methodology, investigation, data curation, validation. **Estevão de Souza Diniz:** methodology, investigation. **Rogério de Brito Aguiar:** investigation, data curation. **Jeam Marcel Geremia:** conceptualization, methodology, investigation, writing – review and editing. **Henrique Mansur:** conceptualization, investigation. **Rita de Cássia Marqueti:** conceptualization, methodology, investigation, writing – review and editing, supervision, resources. **João Luiz Quagliotti Durigan:** conceptualization, methodology, resources, supervision, project administration, writing – review and editing, investigation, funding acquisition, software.

## Ethics Statement

This study was approved by the Institutional Review Board at the University of Brasília (number 68997923.2.0000.8093). Informed consent was obtained in accordance with the Helsinki Declaration and local resolution.

## Conflicts of Interest

The authors declare no conflicts of interest.

## References

[1] D. Ivins , “Acute Ankle Sprain: An Update,” American Family Physician 74, no. 10 (2006): 1714–1720. 17137000

[2] M. M. Herzog , Z. Y. Kerr , S. W. Marshall , and E. A. Wikstrom , “Epidemiology of Ankle Sprains and Chronic Ankle Instability,” Journal of Athletic Training 54, no. 6 (2019): 603–610, 10.4085/1062-6050-447-17. 31135209 PMC6602402

[3] C. Doherty , E. Delahunt , B. Caulfield , J. Hertel , J. Ryan , and C. Bleakley , “The Incidence and Prevalence of Ankle Sprain Injury: A Systematic Review and meta‐Analysis of Prospective Epidemiological Studies,” Sports Medicine 44, no. 1 (2014): 123–140. 24105612 10.1007/s40279-013-0102-5

[4] M. H. Nabian , S. A. Zadegan , L. O. Zanjani , and S. R. Mehrpour , “Epidemiology of Joint Dislocations and Ligamentous/Tendinous Injuries Among 2,700 Patients: Five‐Year Trend of a Tertiary Center in Iran,” Archives of Bone and Joint Surgery 5, no. 6 (2017): 426–434. 29299498 PMC5736892

[5] R. L. Martin , T. E. Davenport , J. J. Fraser , et al., “Ankle Stability and Movement Coordination Impairments: Lateral Ankle Ligament Sprains Revision 2021,” Journal of Orthopaedic and Sports Physical Therapy 51, no. 4 (2021): CPG1–CPG80, 10.2519/jospt.2021.0302. 33789434

[6] R. M. van Rijn , A. G. Os , R. M. Bernsen , P. A. Luijsterburg , B. W. Koes , and S. M. Bierma‐Zeinstra , “What Is the Clinical Course of Acute Ankle Sprains? A Systematic Literature Review,” American Journal of Medicine 121, no. 4 (2008): 324–331, 10.1016/j.amjmed.2007.11.018. 18374692

[7] H. Mansur , M. de Noronha , R. C. Marqueti , and J. L. Q. Durigan , “Acute Lateral Ankle Sprain Alters Muscle and Tendon Properties: Case Series,” Foot and Ankle Surgery 28, no. 3 (2022): 402–408, 10.1016/j.fas.2021.05.008. 34034977

[8] T. J. Hubbard and C. A. Hicks‐Little , “Ankle Ligament Healing After an Acute Ankle Sprain: An Evidence‐Based Approach,” Journal of Athletic Training 43, no. 5 (2008): 523–529, 10.4085/1062-6050-43.5.523. 18833315 PMC2547872

[9] P. A. Gribble , C. M. Bleakley , B. M. Caulfield , et al., “Evidence Review for the 2016 International Ankle Consortium Consensus Statement on the Prevalence, Impact and Long‐Term Consequences of Lateral Ankle Sprains,” British Journal of Sports Medicine 50, no. 24 (2016): 1496–1505, 10.1136/bjsports-2016-096189. 27259753

[10] C. C. Hong , K. J. Tan , and J. Calder , “Chronic Lateral Ankle Ligament Instability ‐ Current Evidence and Recent Management Advances,” Journal of Clinical Orthopaedics and Trauma 48 (2023): 102328, 10.1016/j.jcot.2023.102328. 38274643 PMC10806209

[11] Z. C. Hou , X. Miao , and Y. F. Ao , “Characteristics and Predictors of Muscle Strength Deficit in Mechanical Ankle Instability,” BMC Musculoskeletal Disorders 21, no. 1 (2020): 730, 10.1186/s12891-020-03754-9. 33172443 PMC7654059

[12] C. C. Lobo , C. R. Morales , D. R. Sanz , I. S. Corbalán , A. G. Marín , and D. L. López , “Ultrasonography Comparison of Peroneus Muscle Cross‐Sectional Area in Subjects With or Without Lateral Ankle Sprains,” Journal of Manipulative and Physiological Therapeutics 39, no. 9 (2016): 635–644, 10.1016/j.jmpt.2016.09.001. 27793349

[13] H. Mansur , J. L. Q. Durigan , S. Contessoto , D. A. Maranho , and M. H. Nogueira‐Barbosa , “Evaluation of the Healing Status of Lateral Ankle Ligaments 6 Weeks After an Acute Ankle Sprain,” Journal of Foot and Ankle Surgery 63, no. 6 (2024): 637–645, 10.1053/j.jfas.2024.07.004. 39067610

[14] T. M. Miklovic , L. Donovan , O. A. Protzuk , M. S. Kang , and M. A. Feger , “Acute Lateral Ankle Sprain to Chronic Ankle Instability: A Pathway of Dysfunction,” Physician and Sportsmedicine 46, no. 1 (2018): 116–122, 10.1080/00913847.2018.1409604. 29171312

[15] M. A. Feger , S. Snell , G. G. Handsfield , et al., “Diminished Foot and Ankle Muscle Volumes in Young Adults With Chronic Ankle Instability,” Orthopaedic Journal of Sports Medicine 4, no. 6 (2016): 2325967116653719, 10.1177/2325967116653719. 27570782 PMC4999538

[16] O. R. Seynnes , C. N. Maganaris , M. D. de Boer , P. E. di Prampero , and M. V. Narici , “Early Structural Adaptations to Unloading in the Human Calf Muscles,” Acta Physiologica (Oxford, England) 193, no. 3 (2008): 265–274, 10.1111/j.1748-1716.2008.01842.x. 18266998

[17] E. J. O. Hardy , T. B. Inns , J. Hatt , et al., “The Time Course of Disuse Muscle Atrophy of the Lower Limb in Health and Disease,” Journal of Cachexia, Sarcopenia and Muscle 13, no. 6 (2022): 2616–2629, 10.1002/jcsm.13067. 36104842 PMC9745468

[18] N. D. Reeves , M. V. Narici , and C. N. Maganaris , “Myotendinous Plasticity to Ageing and Resistance Exercise in Humans,” Experimental Physiology 91, no. 3 (2006): 483–498. 16469817 10.1113/expphysiol.2005.032896

[19] S. P. Magnusson and M. Kjaer , “The Impact of Loading, Unloading, Ageing and Injury on the Human Tendon,” Journal of Physiology 597, no. 5 (2019): 1283–1298, 10.1113/JP275450. 29920664 PMC6395417

[20] K. Kubo , H. Akima , J. Ushiyama , et al., “Effects of 20 Days of Bed Rest on the Viscoelastic Properties of Tendon Structures in Lower Limb Muscles,” British Journal of Sports Medicine 38, no. 3 (2004): 324–330, 10.1136/bjsm.2003.005595. 15155437 PMC1724819

[21] K. M. M. E. Lima , J. F. S. Costa Júnior , W. C. A. Pereira , and L. F. Oliveira , “Assessment of the Mechanical Properties of the Muscle‐Tendon Unit by Supersonic Shear Wave Imaging Elastography: A Review,” Ultrasonography 37, no. 1 (2018): 3–15, 10.14366/usg.17017. 28607322 PMC5769952

[22] S. K. Crawford , D. Thelen , J. M. Yakey , B. C. Heiderscheit , J. J. Wilson , and K. S. Lee , “Regional Shear Wave Elastography of Achilles Tendinopathy in Symptomatic Versus Contralateral Achilles Tendons,” European Radiology 33, no. 1 (2023): 720–729, 10.1007/s00330-022-08957-3. 35760909 PMC9771859

[23] X. Zhou , C. Wang , S. Qiu , L. Mao , F. Chen , and S. Chen , “Non‐Invasive Assessment of Changes in Muscle Injury by Ultrasound Shear Wave Elastography: An Experimental Study in a Contusion Model,” Ultrasound in Medicine & Biology 44, no. 12 (2018): 2759–2767, 10.1016/j.ultrasmedbio.2018.07.016. 30172571

[24] R. Kinugasa , J. A. Hodgson , V. R. Edgerton , D. D. Shin , and S. Sinha , “Reduction in Tendon Elasticity From Unloading Is Unrelated to Its Hypertrophy,” Journal of Applied Physiology (1985) 109, no. 3 (2010): 870–877, 10.1152/japplphysiol.00384.2010. PMC2944640 20616227

[25] M. D. de Boer , C. N. Maganaris , O. R. Seynnes , M. J. Rennie , and M. V. Narici , “Time Course of Muscular, Neural and Tendinous Adaptations to 23‐Day Unilateral Lower‐Limb Suspension in Young Men,” Journal of Physiology 583, no. 3 (2007): 1079–1091, 10.1113/jphysiol.2007.135392. 17656438 PMC2277190

[26] J. Hertel , “Sensorimotor Deficits With Ankle Sprains and Chronic Ankle Instability,” Clinics in Sports Medicine 27, no. 3 (2008): 353–370. 18503872 10.1016/j.csm.2008.03.006

[27] I. D. Loram and M. Lakie , “Human Balancing of an Inverted Pendulum: Is Sway Size Controlled by Ankle Impedance?,” Journal of Physiology 540, no. Pt 3 (2002): 1111–1121. 11313453 10.1111/j.1469-7793.2001.0879e.x PMC2278569

[28] K. L. Jakubowski , D. Ludvig , D. Bujnowski , S. S. M. Lee , and E. J. Perreault , “Simultaneous Quantification of Ankle, Muscle, and Tendon Impedance in Humans,” IEEE Transactions on Biomedical Engineering 69, no. 12 (2022): 3657–3666, 10.1109/TBME.2022.3175646. 35594215 PMC10077951

[29] K. L. Jakubowski , D. Ludvig , E. J. Perreault , and S. S. M. Lee , “Non‐Linear Properties of the Achilles Tendon Determine Ankle Impedance Over a Broad Range of Activations in Humans,” Journal of Experimental Biology 226, no. 14 (2023): jeb244863, 10.1242/jeb.244863. 37350252 PMC10399991

[30] T. E. Sakanaka , J. Gill , M. D. Lakie , and R. F. Reynolds , “Intrinsic Ankle Stiffness During Standing Increases With Ankle Torque and Passive Stretch of the Achilles Tendon,” PLoS One 13, no. 3 (2018): e0193850, 10.1371/journal.pone.0193850. 29558469 PMC5860743

[31] K. Chino and H. Takahashi , “Association of Muscle and Tendon Elasticity With Passive Joint Stiffness in the Human Ankle,” European Journal of Applied Physiology 116, no. 5 (2016): 937–944.

[32] I. D. Loram , C. N. Maganaris , and M. Lakie , “The Passive, Human Calf Muscles in Relation to Standing: The Non‐Linear Decrease From Short Range to Long Range Stiffness,” Journal of Physiology 584, no. Pt 2 (2007): 661–675, 10.1113/jphysiol.2007.140046. 17823209 PMC2277155

[33] T. Kobayashi , M. Saka , E. Suzuki , et al., “In Vivo Kinematics of the Talocrural and Subtalar Joints During Weightbearing Ankle Rotation in Chronic Ankle Instability,” Foot & Ankle Specialist 7, no. 1 (2014): 13–19, 10.1177/1938640013514269. 24334366

[34] J. E. Kovaleski , R. J. Heitman , L. R. Gurchiek , J. M. Hollis , W. Liu , and A. W. Pearsall , “Joint Stability Characteristics of the Ankle Complex in Female Athletes With Histories of Lateral Ankle Sprain, Part II: Clinical Experience Using Arthrometric Measurement,” Journal of Athletic Training 49, no. 2 (2014): 198–203, 10.4085/1062-6050-49.2.08. 24568223 PMC3975775

[35] P. Corrigan , J. A. Zellers , P. Balascio , K. G. Silbernagel , and D. H. Cortes , “Quantification of Mechanical Properties in Healthy Achilles Tendon Using Continuous Shear Wave Elastography: A Reliability and Validation Study,” Ultrasound in Medicine & Biology 45, no. 7 (2019): 1574–1585, 10.1016/j.ultrasmedbio.2019.03.015. 31076233 PMC6555647

[36] J. L. Gennisson , C. Cornu , S. Catheline , M. Fink , and P. Portero , “Human Muscle Hardness Assessment During Incremental Isometric Contraction Using Transient Elastography,” Journal of Biomechanics 38, no. 7 (2005): 1543–1550, 10.1016/j.jbiomech.2004.07.013. 15922766

[37] C. L. Liu , Y. X. Zheng , W. H. Zheng , et al., “Muscle Architecture and Mechanical Properties of the Quadriceps and Hamstrings in Athletes With Anterior Cruciate Ligament Reconstruction at 6 Months Post‐Surgery,” International Journal of Sports Medicine 41, no. 3 (2020): 165–174, 10.1055/a-1037-8752.

[38] J. A. Martin , A. H. Biedrzycki , K. S. Lee , et al., “In Vivo Measures of Shear Wave Speed as a Predictor of Tendon Elasticity and Strength,” Ultrasound in Medicine & Biology 41, no. 10 (2015): 2722–2730, 10.1016/j.ultrasmedbio.2015.06.008. 26215492 PMC4556570

[39] T. Mifsud , A. Gatt , K. Micallef‐Stafrace , N. Chockalingam , and N. Padhiar , “Elastography in the Assessment of the Achilles Tendon: A Systematic Review of Measurement Properties,” Journal of Foot and Ankle Research 16, no. 1 (2023): 23, 10.1186/s13047-023-00623-1. 37101290 PMC10134611

[40] L. C. Slane , J. Martin , R. DeWall , D. Thelen , and K. Lee , “Quantitative Ultrasound Mapping of Regional Variations in Shear Wave Speeds of the Aging Achilles Tendon,” European Radiology 27, no. 2 (2017): 474–482, 10.1007/s00330-016-4409-0. 27236815 PMC5125901

[41] S. F. Eby , B. A. Cloud , J. E. Brandenburg , et al., “Shear Wave Elastography of Passive Skeletal Muscle Stiffness: Influences of Sex and Age Throughout Adulthood,” Clinical Biomechanics (Bristol, Avon) 30, no. 1 (2015): 22–27, 10.1016/j.clinbiomech.2014.11.011. 25483294 PMC4298479

[42] E. E. Drakonaki , I. Sudoł‐Szopińska , C. Sinopidis , and P. Givissis , “High‐Resolution Ultrasound for Imaging Complications of Muscle Injury: Is There an Additional Role for Elastography?,” Journal of Ultrasound 19, no. 77 (2019): 137–144, 10.15557/JoU.2019.0020. PMC6750326 31355586

[43] J. M. Geremia , M. F. Bobbert , M. Casa Nova , et al., “The Structural and Mechanical Properties of the Achilles Tendon 2 Years After Surgical Repair,” Clinical Biomechanics (Bristol, Avon) 30, no. 5 (2015): 485–492, 10.1016/j.clinbiomech.2015.03.005. 25828432

[44] M. Goo , L. M. Johnston , F. Hug , and K. Tucker , “Systematic Review of Instrumented Measures of Skeletal Muscle Mechanical Properties: Evidence for the Application of Shear Wave Elastography With Children,” Ultrasound in Medicine & Biology 46, no. 8 (2020): 1831–1840, 10.1016/j.ultrasmedbio.2020.04.009. 32423570

[45] N. Y. Kelp , C. J. Clemente , K. Tucker , F. Hug , S. Pinel , and T. J. M. Dick , “Influence of Internal Muscle Properties on Muscle Shape Change and Gearing in the Human Gastrocnemii,” Journal of Applied Physiology (1985) 134, no. 6 (2023): 1520–1529, 10.1152/japplphysiol.00080.2023. 37167262

[46] W. P. Mayer , J. D. S. Baptista , F. De Oliveira , M. Mori , and E. A. Liberti , “Consequences of Ankle Joint Immobilisation: Insights From a Morphometric Analysis About Fibre Typification, Intramuscular Connective Tissue, and Muscle Spindle in Rats,” Histochemistry and Cell Biology 156, no. 6 (2021): 583–594, 10.1007/s00418-021-02027-3. 34476549

[47] S. Pinel , N. Y. Kelp , J. M. Bugeja , B. Bolsterlee , F. Hug , and T. J. M. Dick , “Quantity Versus Quality: Age‐Related Differences in Muscle Volume, Intramuscular Fat, and Mechanical Properties in the Triceps Surae,” Experimental Gerontology 156 (2021): 111594, 10.1016/j.exger.2021.111594. 34673171

[48] G. K. Thot , C. Berwanger , E. Mulder , et al., “Effects of Long‐Term Immobilisation on Endomysium of the Soleus Muscle in Humans,” Experimental Physiology 106, no. 10 (2021): 2038–2045, 10.1113/EP089734. 34387385

[49] N. Malliaropoulos , E. Papacostas , A. Papalada , and N. Maffulli , “Acute Lateral Ankle Sprains in Track and Field Athletes: An Expanded Classification,” Foot and Ankle Clinics 11, no. 3 (2006): 497–507. 16971243 10.1016/j.fcl.2006.05.004

[50] D. Lacerda , D. Pacheco , A. T. Rocha , P. Diniz , I. Pedro , and F. G. Pinto , “Current Concept Review: State of Acute Lateral Ankle Injury Classification Systems,” Journal of Foot and Ankle Surgery 62, no. 1 (2023): 197–203, 10.1053/j.jfas.2022.08.005. 36184447

[51] Y. E. Gomes , M. Chau , H. A. Banwell , and R. S. Causby , “Diagnostic Accuracy of the Ottawa Ankle Rule to Exclude Fractures in Acute Ankle Injuries in Adults: A Systematic Review and meta‐Analysis,” BMC Musculoskeletal Disorders 23, no. 1 (2022): 885, 10.1186/s12891-022-05831-7. 36151550 PMC9502997

[52] A. Mizusaki Imoto , S. Peccin , R. Rodrigues , and J. Mizusaki , “Translation, Cultural Adaptation and Validation of Foot and Ankle Outcome Score (FAOS) Questionnaire Into Portuguese,” Acta Ortopédica Brasileira 17, no. 4 (2009): 232–235, 10.1590/S1413-78522009000400008.

[53] P. Terrier , S. Piotton , I. M. Punt , J. L. Ziltener , and L. Allet , “Predictive Factors of Recovery After an Acute Lateral Ankle Sprain: A Longitudinal Study,” Sports 9, no. 3 (2021): 41, 10.3390/sports9030041. 33803881 PMC8003324

[54] N. Devoogdt and C. Cavaggion , “Reliability, Validity, and Feasibility of Water Displacement Method, Figure‐Of‐Eight Method, and Circumference Measurements in Determination of Ankle and Foot Edema,” Lymphatic Research and Biology 17, no. 5 (2019): 531–536. 30648912 10.1089/lrb.2018.0045

[55] H. P. Smitheman , K. D. Seymore , M. N. Potter , A. K. Smith , S. Aufwerber , and K. G. Silbernagel , “Measurement of Healthy and Injured Triceps Surae Morphology,” Journal of Visualized Experiments 200 (2023): e65798, 10.3791/65798. PMC10860649 37955382

[56] G. Ferraioli , R. G. Barr , A. Farrokh , et al., “How to Perform Shear Wave Elastography. Part II,” Medical Ultrasonography 24, no. 2 (2022): 196–210, 10.11152/mu-3342. 34379714

[57] R. Ando and Y. Suzuki , “Positive Relationship Between Passive Muscle Stiffness and Rapid Force Production,” Human Movement Science 66 (2019): 285–291, 10.1016/j.humov.2019.05.002. 31082668

[58] G. Dubois , W. Kheireddine , C. Vergari , et al., “Reliable Protocol for Shear Wave Elastography of Lower Limb Muscles at Rest and During Passive Stretching,” Ultrasound in Medicine & Biology 41, no. 9 (2015): 2284–2291, 10.1016/j.ultrasmedbio.2015.04.020. 26129731

[59] C. Ewertsen , J. F. Carlsen , I. R. Christiansen , J. A. Jensen , and M. B. Nielsen , “Evaluation of Healthy Muscle Tissue by Strain and Shear Wave Elastography ‐ Dependency on Depth and ROI Position in Relation to Underlying Bone,” Ultrasonics 71 (2016): 127–133, 10.1016/j.ultras.2016.06.007. 27336792

[60] J. Ryu and W. K. Jeong , “Current Status of Musculoskeletal Application of Shear Wave Elastography,” Ultrasonography 36, no. 3 (2017): 185–197, 10.14366/usg.16053. 28292005 PMC5494870

[61] J. Cohen , “The Earth Is Round (p<. 05): Rejoinder,” American Psychologist 50, no. 12 (1995): 1103, 10.1037/0003-066X.50.12.1103.

[62] L. G. Portney and M. P. Watkins , Foundations of Clinical Research: Applications to Practice, 2nd ed. (Prentice Hall Health, 2000).

[63] C. Doherty , C. Bleakley , J. Hertel , B. Caulfield , J. Ryan , and E. Delahunt , “Lower Extremity Function During Gait in Participants With First‐Time Acute Lateral Ankle Sprain Compared to Controls,” Journal of Electromyography and Kinesiology 25, no. 1 (2015): 182–192, 10.1016/j.jelekin.2014.09.004. 25443172

[64] B. C. Clark , N. K. Mahato , M. Nakazawa , T. D. Law , and J. S. Thomas , “The Power of the Mind: The Cortex as a Critical Determinant of Muscle Strength/Weakness,” Journal of Neurophysiology 112, no. 12 (2014): 3219–3226, 10.1152/jn.00386.2014. 25274345 PMC4269707

[65] S. P. Kilroe , J. Fulford , S. R. Jackman , L. J. C. VAN Loon , and B. T. Wall , “Temporal Muscle‐Specific Disuse Atrophy During One Week of Leg Immobilization,” Medicine and Science in Sports and Exercise 52, no. 4 (2020): 944–954, 10.1249/MSS.0000000000002200. 31688656

[66] B. Christensen , E. Dyrberg , P. Aagaard , M. Kjaer , and H. Langberg , “Short‐Term Immobilization and Recovery Affect Skeletal Muscle but Not Collagen Tissue Turnover in Humans,” Journal of Applied Physiology (1985) 105, no. 6 (2008): 1845–1851, 10.1152/japplphysiol.90445.2008. 18927270

[67] S. I. Docking and J. Cook , “How Do Tendons Adapt? Going Beyond Tissue Responses to Understand Positive Adaptation and Pathology Development: A Narrative Review,” Journal of Musculoskeletal & Neuronal Interactions 19, no. 3 (2019): 300–310. 31475937 PMC6737558

[68] L. N. Zhang , W. B. Wan , Y. X. Wang , et al., “Evaluation of Elastic Stiffness in Healing Achilles Tendon After Surgical Repair of a Tendon Rupture Using In Vivo Ultrasound Shear Wave Elastography,” Medical Science Monitor 22 (2016): 1186–1191, 10.12659/MSM.895674. 27072885 PMC4835154

[69] K. Yoshida , Y. Itoigawa , Y. Maruyama , and K. Kaneko , “Healing Process of Gastrocnemius Muscle Injury on Ultrasonography Using B‐Mode Imaging, Power Doppler Imaging, and Shear Wave Elastography,” Journal of Ultrasound in Medicine 38, no. 12 (2019): 3239–3246, 10.1002/jum.15035. 31165497

[70] Z. Wang , G. Lyu , H. Zhong , L. Yan , and Z. Xu , “Shear Wave Elastography for Detecting Calf Muscle Stiffness: An Effective Tool for Assessing Sarcopenia,” Journal of Ultrasound in Medicine 42, no. 4 (2023): 891–900, 10.1002/jum.16082. 36000347

[71] X. M. Chen , L. G. Cui , P. He , W. W. Shen , Y. J. Qian , and J. R. Wang , “Shear Wave Elastographic Characterization of Normal and Torn Achilles Tendons: A Pilot Study,” Journal of Ultrasound in Medicine 32, no. 3 (2013): 449–455, 10.7863/jum.2013.32.3.449. 23443185

[72] B. Frankewycz , A. Penz , J. Weber , et al., “Achilles Tendon Elastic Properties Remain Decreased in Long Term After Rupture,” Knee Surgery, Sports Traumatology, Arthroscopy 26, no. 7 (2018): 2080–2087, 10.1007/s00167-017-4791-4. 29147741

[73] H. Zhang , W. Peng , C. Qin , et al., “Lower Leg Muscle Stiffness on Two‐Dimensional Shear Wave Elastography in Subjects With Medial Tibial Stress Syndrome,” Journal of Ultrasound in Medicine 41, no. 7 (2022): 1633–1642, 10.1002/jum.15842. 34617298

[74] A. Busilacchi , M. Olivieri , S. Ulisse , et al., “Real‐Time Sonoelastography as Novel Follow‐Up Method in Achilles Tendon Surgery,” Knee Surgery, Sports Traumatology, Arthroscopy 24, no. 7 (2016): 2124–2132, 10.1007/s00167-014-3484-5. 25539686

[75] P. Corrigan , D. H. Cortes , R. T. Pohlig , and K. Grävare Silbernagel , “Tendon Morphology and Mechanical Properties Are Associated With the Recovery of Symptoms and Function in Patients With Achilles Tendinopathy,” Orthopaedic Journal of Sports Medicine 8, no. 4 (2020): 2325967120917271, 10.1177/2325967120917271. 32426410 PMC7218994

[76] D. H. Cortes , S. M. Suydam , K. G. Silbernagel , T. S. Buchanan , and D. M. Elliott , “Continuous Shear Wave Elastography: A New Method to Measure Viscoelastic Properties of Tendons In Vivo,” Ultrasound in Medicine & Biology 41, no. 6 (2015): 1518–1529, 10.1016/j.ultrasmedbio.2015.02.001. 25796414 PMC4426016

[77] T. Dirrichs , V. Quack , M. Gatz , M. Tingart , C. K. Kuhl , and S. Schrading , “Shear Wave Elastography (SWE) for the Evaluation of Patients With Tendinopathies,” Academic Radiology 23, no. 10 (2016): 1204–1213, 10.1016/j.acra.2016.05.012. 27318786

[78] S. L. Hanlon , R. Scattone Silva , B. J. Honick , and K. G. Silbernagel , “Effect of Symptom Duration on Injury Severity and Recovery in Patients With Achilles Tendinopathy,” Orthopaedic Journal of Sports Medicine 11, no. 5 (2023): 23259671231164956, 10.1177/23259671231164956. 37250747 PMC10214069

[79] K. D. Seymore , H. P. Smitheman , A. K. Smith , R. T. Pohlig , C. Couppé , and K. G. Silbernagel , “Metabolic Risk Factors Relate to Worse Tendon Health in Individuals With Achilles Tendinopathy,” Journal of Orthopaedic Research 43 (2025): 728–738, 10.1002/jor.26038. 39763090 PMC11903168

[80] M. Kawai , K. Taniguchi , T. Suzuki , and M. Katayose , “Estimation of Quadriceps Femoris Muscle Dysfunction in the Early Period After Surgery of the Knee Joint Using Shear‐Wave Elastography,” BMJ Open Sport & Exercise Medicine 4, no. 1 (2018): e000381, 10.1136/bmjsem-2018-000381. PMC6196956 30364553

[81] A. L. McPherson , N. A. Bates , C. R. Haider , T. Nagai , T. E. Hewett , and N. D. Schilaty , “Thigh Musculature Stiffness During Active Muscle Contraction After Anterior Cruciate Ligament Injury,” BMC Musculoskeletal Disorders 21, no. 1 (2020): 320, 10.1186/s12891-020-03342-x. 32438905 PMC7243327

[82] R. M. Khair , M. Sukanen , and T. Finni , “Achilles Tendon Stiffness: Influence of Measurement Methodology,” Ultrasound in Medicine & Biology 50, no. 10 (2024): 1522–1529, 10.1016/j.ultrasmedbio.2024.06.005. 39079832

[83] F. M. Gonzalez , C. A. Gleason , K. S. Lee , et al., “Shear Wave Elastography Assessment and Comparison Study of the Achilles Tendons in Optimally Conditioned Asymptomatic Young Collegiate Athletes,” Skeletal Radiology 50, no. 12 (2021): 2381–2392, 10.1007/s00256-021-03798-5. 33963895

[84] M. A. Pelea , O. Serban , M. Badarinza , R. Gutiu , and D. Fodor , “Shear‐Wave Elastography of the Achilles Tendon: Reliability Analysis and Impact of Parameters Modulating Elasticity Values,” Journal of Ultrasound 27, no. 3 (2024): 559–566, 10.1007/s40477-024-00877-w. 38613661 PMC11333681

